# Towards Controlling the Glycoform: A Model Framework Linking Extracellular Metabolites to Antibody Glycosylation

**DOI:** 10.3390/ijms15034492

**Published:** 2014-03-14

**Authors:** Philip M. Jedrzejewski, Ioscani Jimenez del Val, Antony Constantinou, Anne Dell, Stuart M. Haslam, Karen M. Polizzi, Cleo Kontoravdi

**Affiliations:** 1 Centre for Process Systems Engineering, Department of Chemical Engineering, Imperial College London, London SW7 2AZ, UK; E-Mails: philip.jedrzejewski07@imperial.ac.uk (P.M.J.); ioscani.jimenez-del-val06@imperial.ac.uk (I.J.V.); 2 Department of Life Sciences, Imperial College London, London SW7 2AZ, UK; E-Mails: a.constantinou@imperial.ac.uk (A.C.); a.dell@imperial.ac.uk (A.D.); s.haslam@imperial.ac.uk (S.M.H.); k.polizzi@imperial.ac.uk (K.M.P.); 3 Centre for Synthetic Biology and Innovation, Imperial College London, London SW7 2AZ, UK

**Keywords:** metabolic modelling, nucleotide sugars, antibody glycosylation

## Abstract

Glycoproteins represent the largest group of the growing number of biologically-derived medicines. The associated glycan structures and their distribution are known to have a large impact on pharmacokinetics. A modelling framework was developed to provide a link from the extracellular environment and its effect on intracellular metabolites to the distribution of glycans on the constant region of an antibody product. The main focus of this work is the mechanistic *in silico* reconstruction of the nucleotide sugar donor (NSD) metabolic network by means of 34 species mass balances and the saturation kinetics rates of the 60 metabolic reactions involved. NSDs are the co-substrates of the glycosylation process in the Golgi apparatus and their simulated dynamic intracellular concentration profiles were linked to an existing model describing the distribution of *N*-linked glycan structures of the antibody constant region. The modelling framework also describes the growth dynamics of the cell population by means of modified Monod kinetics. Simulation results match well to experimental data from a murine hybridoma cell line. The result is a modelling platform which is able to describe the product glycoform based on extracellular conditions. It represents a first step towards the *in silico* prediction of the glycoform of a biotherapeutic and provides a platform for the optimisation of bioprocess conditions with respect to product quality.

**Table:** Nomenclature

*V*	culture volume	L
*X*_V_	cell density	cells/L
*t*	time	h
μ	cell growth rate	h^−1^
*k*_d_	cell death rate	h^−1^
*F*_out_	flow rate out of culture	L/h
Glc_ext_	extracellular glucose concentration	mM
Gln_ext_	extracellular glutamine concentration	mM
*F*_in_	flow rate into culture	L/h
Glc_feed_	feed glucose concentration	mM
*K*_A_	activator species saturation coefficient	mM
*K*_M_	species saturation coefficient	mM
*k*_d,max_	maximum cell death rate	h^−1^
*K*_d_	species depletion coefficient	mM
*q*	species cellular production	mmol/(h-cell)
*Y*	species yield	cell/mmol
*m*	species cell maintenance term	mmol/(h-cell)
mAb	Antibody product titer	mM
Nuc	Intracellular nucleotide concentration	mM
*K*_TP_	Transport protein species saturation coefficient	mM
*V*_cell_	cell volume	L
DNA_f_	nucleotide fraction in DNA	dimensionless
*m*_DNA_	cellular DNA mass	mg/cell
*m*_RNA_	cellular RNA mass	mg/cell
Mr	molecular species mass	mg/mmol
RNA_f_	nucleotide fraction in RNA	dimensionless
*k*_cat_	enzyme turnover rate	h^−1^
*K*_i_	Species inhibition constant	mM
*E*_0_	Initial enzyme concentration	mM
*N*_gly,cell_	Number of glycans per cell	mmol/cell
*N*_NSD,gly_	NSDs consumed per host cell glycan	mmol/mmol
*N*_gly,mAb_	Number of glycans per antibody	mmol/mmol
*N*_NSD,mAb_	NSDs consumed per antibody	mmol/mmol
*F*_mAb_	Antibody production rate	mmol/h

## 1. Introduction

Biotechnological products, including bioengineered vaccines and recombinant proteins, constitute 19% of the pharmaceutical market with a total sales value of $142bn as of 2011 and substantial anticipated growth [1]. Glycoproteins represent the largest group of biologically-derived medicines, constituting 78 out of 212 approved products by the EMA as of May 2012 [2]. *N*-linked glycans exert major influence over the *in vivo* therapeutic mechanisms of the protein and have attracted much industrial interest. For example, with monoclonal antibodies, the largest class of biotherapeutic glycoproteins [2], the absence of the core fucose on the conserved Asn297 in the Fc region can increase antibody-dependent cell-mediated cytotoxicity (ADCC) up to 50-fold [3,4]. Galactose terminating structures are known to have a substantial effect on the affinity towards the C1q complex and their removal results in decreased complement lysis activity [5]. Other examples are 2,6-linked terminating sialic acid glycan motifs, which enable antibodies to modulate anti-inflammatory immune response [6] and high mannose content, which can lead to reduced *in vivo* half-life [7]. These findings have quickly been translated into the development of third generation mAbs [8]. Notable impact of glycans and the glycoform on the performance of biotherapeutics is not only limited to antibodies but also extends to other industrially relevant glycoproteins such as IFN-β and EPO amongst others [9].

### Current Problems Resulting from Glycans and Causes of Variation

A well-defined product may have consistent protein backbones but can still display a glycoform distribution of more than a hundred detectable isoforms [10]. This is still a small proportion of all the potential glycan structures (estimated to be in excess of 20,000 potential structures) [11]. Protein *N*-glycosylation begins when a glycan precursor structure is attached to the protein backbone in the endoplasmic reticulum. The glycoprotein is subsequently transported to the Golgi apparatus where the high-mannose glycans are enzymatically processed giving rise to large variations in glycoform depending on host cell line, enzyme concentrations/availability, various process conditions, and the availability of the reaction substrates, *i.e.*, nucleotide sugar donor species (NSDs) (extensively discussed in [12]). The nucleotide sugars donors are synthesized in the cell cytoplasm subject to process conditions and are then transported into the Golgi. This opens the door for engineering of the glycoform by means of feeding strategies, where the addition of specific metabolic intermediates of the nucleotide sugar synthesis pathways to the culture will drive metabolic flux towards the desired nucleotide sugar and eventually influence the glycoform through increased substrate availability [13,14]. Many sugar and/or nucleotide feeding strategies have been successfully pursued in order to manipulate the glycoform of glycoproteins (summarized previously [9,15]). However, the predictions of the impact of feeding strategies onto the glycoform still remain qualitative, a gap that can most accurately be filled through a modeling framework.

Under the information-driven Quality by Design (QbD) paradigm, upfront experimentation is increased to gain a better understanding of the factors effecting critical Quality Attributes (cQAs) such as the effect of NSD species availability on the final *N*-linked glycoform [16]. Based on experimentally generated data and available biological knowledge, modelling frameworks can be formulated to recreate biological systems such as metabolic networks and their responses to perturbations. This, in turn, can aid the design and optimisation of the biotherapeutic manufacturing process with respect to both product titer and quality.

In this work, the nucleotide sugar donor metabolic synthesis network was recreated *in silico.* The structure was based on available knowledge of the nucleotide and NSD synthesis pathways and parameters were estimated from experimentally generated data from a hybridoma cell culture system producing an IgG1 monoclonal antibody. Flux-based constraints were also applied to the mechanistic model to enable an improved estimation of unknown parameters. The dynamic outputs of the *in silico* metabolic network were subsequently used to estimate the glycoform of the Fc region of the antibody using a model for *N*-linked glycosylation developed by del Val *et al.* [17]. The results show that the model framework is able to describe the behavior of this cell line, its nucleotide and nucleotide sugar metabolic network as well as the product glycoform.

## 2. Mathematical Model Development

**Figure 1.**
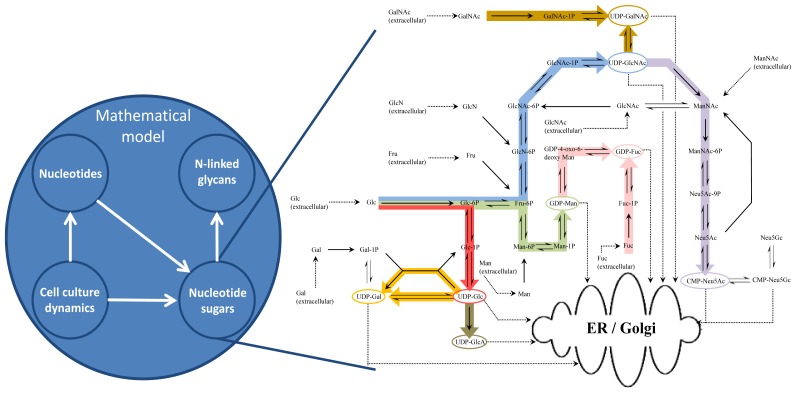
Structure of the mathematical model and the interactions of the individual model parts with a focus on the network for nucleotide sugar synthesis.

The aim of the mathematical modelling framework presented herein is the estimation of intracellular nucleotide sugar concentrations based on extracellular glucose and glutamine levels, with an ultimate goal of predicting the impact of feeding strategies on glycoform distribution. The different parts of the framework and their interactions are shown in Figure 1. It comprises an unstructured cell growth model, and kinetic models of nucleotide and nucleotide sugar synthesis, which finally link to the model describing *N*-linked glycosylation in monoclonal antibodies [17]. The main focus of this work is on a bottom-up modelling approach for the *in silico* reconstruction of the nucleotide sugar donor (NSD) synthesis metabolic network. This approach implies that the system is constructed from its individual components to give rise to a complex model, which translates to describing all known reactions in full dynamic detail to represent the NSD model. The NSD part of the modelling framework uses glucose inlet flux as its sole input and has outputs for glycolysis and the NSD transport to the ER and Golgi.

### 2.1. Cell Culture Dynamics Model

The dynamic cell growth model describes cell culture dynamics as a function of extracellular glucose and glutamine availability. It is based on Monod kinetics with modifications in order to account for non-ideal effects observed in cell culture experiments. Homogeneity with respect to species and cell concentrations was assumed at all times. The viable cell density (*X_V_* in cells/L) is a function of growth rate (μ in h^−1^), death rate (*k*_d_ in h^−1^) and the flow out of the reactor (*F*_out_ in L/h):

(1) $$\frac{dVX_{V}}{dt}=\mu VX_{V}-k_{d}VX_{V}-F_{out}X_{V}$$

The growth rate is a function of extracellular glucose (Glc), glutamine (Gln), the saturation coefficients for glucose (*K*_M,glc_) and glutamine (*K*_M,gln_) all in units of mMol and the maximum growth rate μ_max_ (h^−1^).

(2) $$\mu =\mu_{max}\;\left(\frac{\left[{Glc}_{ext}\right]}{K_{M,glc}+[{Glc}_{ext}]}\right)\;\left(\frac{\left[{Gln}_{ext}\right]}{K_{M,gln}+[{Gln}_{ext}]}\right)$$

The death rate is an inverse Monod function of the glucose and glutamine availability, the maximum death rates for glucose (*k*_d,max,glc_ in h^−1^) and glutamine (*k*_d,max,gln_ in h^−1^), the species depletion coefficients for glucose (*K*_M,gln_ in mmol) and glutamine (*K*_M,gln_ in mmol). Parameter estimation using the gPROMS model building environment [18] showed that extracellular ammonia and lactate did not contribute significantly towards cell death or growth inhibition for this hybridoma cell line (not shown). Therefore, neither species features in other parts of the model.

(3) $$k_{d}=k_{d,max,glc}\;\left(\frac{K_{d,glc}}{K_{d,glc}+[{Glc}_{ext}]}\right)+k_{d,max,gln}\;\left(\frac{K_{d,gln}}{K_{d,gln}+[{Gln}_{ext}]}\right)$$

The cell metabolism of glucose and glutamine were represented by the following equations:

(4) $$\frac{dV[{Glc}_{ext}]}{dt}=-q_{glc}VX_{V}-F_{in}[{Glc}_{feed}]-F_{out}[{Glc}_{ext}]$$

(5) $$\frac{dV[{Gln}_{ext}]}{dt}=-q_{gln}VX_{V}-F_{in}[{Gln}_{feed}]-F_{out}[{Gln}_{ext}]$$

In the above, *q*_glc_ and *q*_gln_ (mmol/(h-cell)) denote the specific glucose and glutamine cell uptake rate, respectively, and are defined by the equations shown below:

(6) $$q_{glc}=\left(\frac{\mu }{Y_{\text{X}v/glc}}\right)+m_{glc}$$

(7) $$q_{gln}=\left(\frac{\mu }{Y_{\text{X}v/gln}}\right)+m_{gln}$$

*Y_Xv_*_/glc_ and *Y_Xv_*_/gln_ (cell/mmol) denote the biomass yield coefficient for glucose and glutamine, respectively. The *m*_glc_ and *m*_gln_ (mmol/(h-cell)) terms are the maintenance coefficients for glucose and glutamine, respectively. Lastly the product term and the specific productivity terms are represented by the following:

(8) $$\frac{dV[mAb]}{dt}=q_{mAb}VX_{V}-F_{out}[mAb]$$

(9) $$q_{mAb}=Y_{mAb/Xv}$$

### 2.2. Nucleotide Model

**Figure 2.**
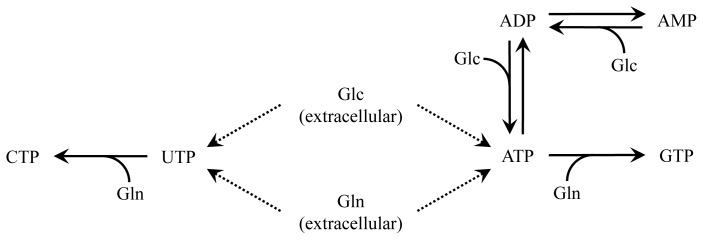
Reduced nucleotide synthesis network.

A nucleotide model was created based on a simplified purine and pyrimidine synthesis network. The carbon source for the synthesis of the nucleotide pentoses is glucose with glutamine acting as the nitrogen source [19], while asparagine and glycine are excluded due to lack of experimental data. The resulting network contains six mass balances, one each for ADP, AMP, ATP, CTP, GTP and UTP connected by a network of eight reactions based on Monod kinetics as displayed in Figure 2. All glucose and glutamine co-substrate terms are based on extracellular concentrations of the two species. The reactions of ATP to ADP and ADP to AMP are the following:

(10) $$r=k\;\left(\frac{\left[Nuc\right]}{K_{M,nuc}+[Nuc]}\right)$$

The synthesis of CTP and GTP are represented by the following equation:

(11) $$r=k\;\left(\frac{\left[Nuc\right]}{K_{M,nuc}+[Nuc]}\right)\times \left(\frac{\left[{Gln}_{ext}\right]}{K_{M,gln}+[{Gln}_{ext}]}\right)$$

The reaction of AMP to ADP and ADP to ATP are of the following form:

(12) $$r=k\;\left(\frac{\left[Nuc\right]}{K_{M,nuc}+[Nuc]}\right)\times \left(\frac{\left[{Glc}_{ext}\right]}{K_{M,glc}+[{Glc}_{ext}]}\right)$$

Lastly, the synthesis of ATP and UTP are represented by the following:

(13) $$r=k\;\left(\frac{\left[{Gln}_{ext}\right]}{K_{M,gln}+[{Gln}_{ext}]}\right)\times \left(\frac{\left[{Glc}_{ext}\right]}{K_{M,glc}+[{Glc}_{ext}]}\right)$$

Extracellular glucose and glutamine are the sole inlets into the nucleotide model. There are a total of four flux-constrained outlets, one each for AMP, CTP, GTP and UTP. For the outlets, it was assumed that each species is consumed in the formation of NSDs for host cell glycoproteins as well as antibody production, DNA synthesis and RNA synthesis. The latter two were taken to be dependent on growth rate and are described by the following equation:

(14) $$F_{out,nuc}=\left(\frac{\left[Nuc\right]}{K_{TP,nuc}+[Nuc]}\right)\times \frac{\mu }{V_{cell}}\times \left(\frac{{DNA}_{f,nuc}m_{DNA}}{{Mr}_{DNA}}+\frac{{RNA}_{f,nuc}m_{RNA}}{{Mr}_{RNA}}\right)$$

The molecular mass for each DNA monomer (*Mr*_DNA_) and RNA monomer (*Mr*_RNA_) as well as the fraction of each nucleotide in DNA (DNA_f,nuc_) and RNA (RNA_f,nuc_) was based on the data used by Nolan and Lee [20]. The mass of DNA (*m*_DNA_) and RNA (*m*_RNA_) were taken as 7.05 and 28.55 pg/cell, respectively [21].

### 2.3. Nucleotide Sugar Synthesis Model

**Figure 3.**
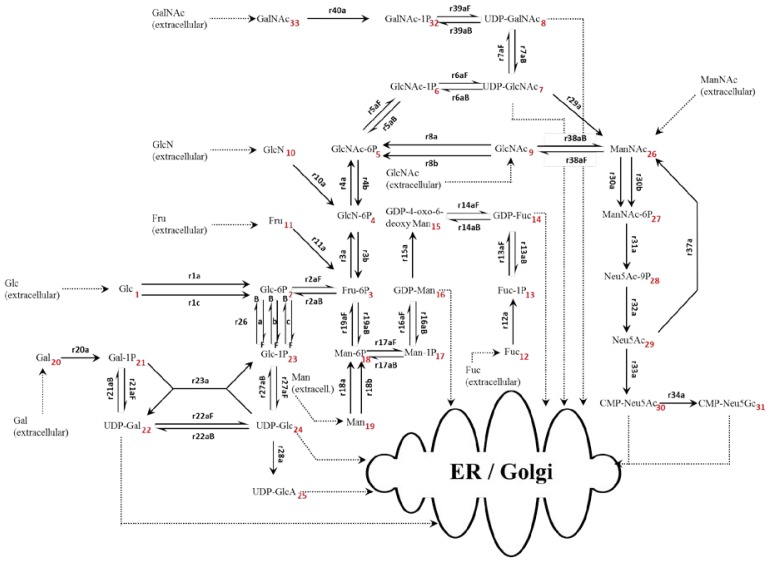
Reaction network including all steps considered in the model.

The bottom-up reconstruction of the NSD metabolic network is based on the murine metabolic network as depicted in the Kyoto Encyclopedia of Genes and Genomes [22] and individual enzyme data as found on BRENDA [23]. In total, the network includes 34 sugar and nucleotide sugar species, each represented as a separate mass balance connected through a network of 35 reaction steps as shown in Figure 3. The rate of each enzymatic reaction is represented by a single equation and is used to predict the dynamic behavior of the metabolic network with respect to the system inputs, *i.e.*, hexoses and nucleotides.

Individual rate expressions are based on enzyme mechanisms and kinetics found in the literature and are summarized in the supplementary information together with dissociation constants (*K*_m_), turnover rates (*k*_cat_), inhibitory terms (*K*_i_), enzyme activity and any Hill coefficients (Table S1). Rate equations are based on Michaelis-Menten kinetics, accounting for reported reaction mechanisms and inhibitory compounds and the Hill equation is used where data indicating deviation from Michaelis-Menten kinetics (*i.e.*, independent binding) was available. Murine (*Mus musculus*) enzyme data was used where available and when lacking preference in data was based on most recent common ancestors as described in the molecular tree of mammals of the placental orders [24]. While the dissociation constant (*K*_m_) is an intrinsic parameter, *V*_max_ is not, and is a function of the turnover rate constant, *k*_cat_, and the initial enzyme concentration used in a particular experimental condition [*E*_0_]. The following assumptions were made for the derivation of the rate of reaction expressions:

- Equilibrium is rapidly reached for all intermediate reactants;
- Rate-limiting steps are irreversible (Table S1);
- Where water is required for catalysis, full enzyme saturation is assumed due to the aqueous environment of the cytoplasm;
- Where more than one substrate is required for catalysis, a random order of substrate binding is assumed, unless reported otherwise;
- Rapid dissociation of reaction products from enzyme;
- Michaelis-Menten kinetics are assumed to hold true, unless reported otherwise;
- All enzyme and transport protein concentrations throughout the network are constant.

In total the mechanisms split into 20 single substrate uni-uni reactions, seven random order bi-bi, 14 ordered bi-bi, 17 Ping-Pong bi-bi and one Ping-Pong ter-ter. The model equations for the most simple uninhibited reaction case and the single specific ter-ter mechanism equations are shown below. A and B denote substrate concentrations, *k*_cat_ turnover rate, *E*_0_ initial enzyme concentration, and I denotes inhibitor concentrations. The derivations of rate of reaction equations for each type of mechanism including inhibitory terms are summarized more extensively in the supplementary information (Appendix 1). Each individual reaction and its corresponding mechanism are also listed in the supplementary information (Table S1). The equations for each type of kinetics are shown below.

Single substrate uni-uni enzyme kinetics:

(15) $$v=\frac{d[P]}{dt}=\frac{k_{cat}[E_{0}][A]}{K_{m}+[A]}$$

Random order bi-bi enzyme kinetics:

(16) $$v=\frac{d[P]}{dt}=\frac{k_{cat}[E_{0}][A][B]}{K_{m,A}K_{m,B}+K_{m,A}[B]+K_{m,B}[A]+[A][B]}$$

Ordered bi-bi enzyme kinetics:

(17) $$v=\frac{d[P]}{dt}=\frac{k_{cat}[E_{0}][A][B]}{K_{m,A}K_{m,B}+K_{m,B}[A]+[A][B]}$$

Ping-pong bi-bi enzyme kinetics:

(18) $$v=\frac{d[P]}{dt}=\frac{k_{cat}[E_{0}][A][B]}{K_{m,A}[B]+K_{m,B}[A]+[A][B]}$$

Ping-pong ter-ter enzyme kinetics:

(19) $$v=\frac{d[P]}{dt}=\frac{k_{cat}[E_{0}][A]{\left[B\right]}^{2}}{2K_{m,A}K_{m,B}[B]+2K_{m,B}[A][B]+K_{m,A}{\left[B\right]}^{2}\;\left(1+\frac{\left[I\right]}{K_{I}}\right)+{K_{m,B}}^{2}[A]+[A]{\left[B\right]}^{2}}$$

The reactions of the NSD network include five reactions with allosteric regulation, six species with cooperative binding behavior and a total of 76 competitive, non-competitive and un-competitive inhibitory terms. For the case of enzyme activation through allosteric regulation, the initial enzyme concentration term was replaced as shown below.

(20) $$[E_{0}]=\frac{\left[E_{0}][A_{activator}\right]}{K_{A,A}+[A_{activator}]}$$

Additionally, there are nine transport rate equations, each accounting for the transport of a specific nucleotide sugar species out of the model, *i.e.*, from the cytoplasm into the Golgi or ER. The transport equations contain a Michaelis-Menten kinetics term including the transport protein dissociation constants, as summarized by del Val *et al.* [17], and a second term reflecting the theoretical nucleotide sugar consumption rate. An exception to this was the CMP-Neu5Ac transporter, which is known to be competitively inhibited by UDP-HexNAc species and hence, an inhibitory term has been included in the transport protein dissociation constant to reflect this [25].

(21) $$NSD\;transport\;rate=\left(\frac{\left[NSD\right]}{K_{TP,NSD}+[NSD]}\right)\;\left[\left(\frac{\mu }{V_{cell}}N_{gly,cell}N_{NSD,gly}\right)+\left(N_{gly,mAb}N_{NSD,mAb}F_{mAb}\right)\right]$$

The consumption rate contains terms for the host cell glycan structures and the antibody product. The first term of the summation describes the rate of glycan addition to host cell protein and is a function of cell growth. The glycan distribution on host cell proteins is assumed to be constant throughout the culture. Based on a protein weight of 33.7 μg/10^5^ cells as reported by Bonarius *et al.* [21] and an average amino acid monomer molecular weight of 127 μg/μmol as calculated by Nolan and Lee [20], an amino acid concentration per cell was found. Multiplying the cellular amino acid concentration with an *O*-linked glycan frequency of 0.00371 glycans per amino and an *N*-linked glycan frequency of 0.00400 glycans per amino acid for 64.9% of glycosylated host cell proteins [26], an *O*-linked and *N*-linked glycan concentration of 1.41 × 10^−8^ and 1.31 × 10^−8^ μmol/cell, respectively, was calculated for each glycan species. This in turn gives a total number of glycans per cell of 2.72 × 10^−8^ μmol/cell (*N*_glyc,cell_). Furthermore, mass spectrometry data of the human mature B-cell glycoform was used to find the average compositions of *O*-linked and *N*-linked glycan structures based on the relative abundance of each species. The findings for each sugar species are summarized in Table 1 and the abundance of each individual identified species as well as the MS methodology on which the average glycoform are based are shown in Appendix 2. Thus, the system becomes constrained through the nine calculated outlet fluxes required to maintain cell growth. The glucose flux towards glycolysis is a function of the enzyme concentration (*E*_glyc_) for the reaction step from fructose 6-phosphate to fructose 1,6-bisphosphate and thus, is allowed to vary as part of the parameter estimation as well as with the concentration of its substrate fructose 6-phosphate. The kinetic data and reaction mechanism were obtained from Chen *et al.* [27]. This step is assumed to be irreversible and acts as the main outlet flux from the NSD network.

The antibody term is based on the antibody production rate from the cell growth model and the experimentally determined glycoform of the HFN7.1 monoclonal antibody.

While enzyme concentration levels of the nucleotide sugar metabolic network have been predicted to change based on transcriptomic analysis [28], they were held constant as part of this study due to lack of dynamic proteomic data. An initial enzyme concentration value was obtained based on an average hybridoma cell protein dry weight fraction and an averaged cell volume as reported by Frame *et al.* [29] and Lee *et al.* [29,30]. The average enzyme purification was found to be 1000-fold with an average molecular mass of 61 kDa based on the obtained literature values listed in the supplementary information (Table S1). Each protein was assumed to make up 0.1% of total cell protein resulting in an average molar enzyme concentration of 3.9 μM, which was used as an initial value for all subsequent parameter estimations.

Glycolysis and the pentose phosphate pathway account for the majority of glucose and other hexose feed consumption in mammalian cells. The first three species of the nucleotide sugar network, namely glucose, glucose-6-phosphate and fructose-6-phosphate are substrates for both glycolysis and nucleotide sugar synthesis and, hence, the fraction consumed by glycolysis needs to be accounted for.

Studies by Fujita *et al.* on phosphomannose isomerase deficient mice suggested that the mannose salvage pathway is in fact substantial [31]. As a result the cleaved mannose from the glycan core structure is fed back into the metabolic pathway as part of the mathematical model. The amount of salvaged mannose per *N*-linked glycan is obtained through the difference of the original glycan precursor structure featuring nine mannose molecules and the final average mannose count per glycan of the host cell protein or antibody product. This also applies to UDP-Glc, where the glucose molecules added to the glycan precursor are salvaged as intracellular glucose. It was assumed that four UDP-Glc molecules are consumed per glycan in the Calnexin and Calreticulin cycle.

Including reverse and parallel reactions as well as the nine transport rates, a total of 69 distinct reactions are simulated as part of the bottom-up approach model. In addition, there are nine non-NSD species held at constant concentration (Appendix 3) and intracellular glutamine, which due to lack of experimental data has been assumed to follow intracellular glucose concentrations. While initial concentrations for nucleotide sugar species have been experimentally determined, no data was available for any intermediate species. The initial concentrations of the remaining 27 species have been estimated at steady state using the mathematical model such that the initial conditions for the seven experimentally measureable nucleotide sugars are met.

### 2.4. Parameter Estimation

A total of 46 parameters were initially estimated using gPROMS to give a first fit for the experimentally derived data. In a second step, a global sensitivity analysis was performed to reduce the number of estimated parameters. Sensitivity analysis is a powerful tool, which can be used in model development for identifying parameter importance with respect to model outputs. It allows for parameters to be ranked with respect to impact on an output, *i.e.*, the concentration of NSDs. Knowledge about the relationship of model inputs with respect to outputs can offer valuable insights into complex models and thus allow for improved design of experiments [32] or, as presented in this study, allow for more targeted parameter estimation and reduction in model size. The method employed in this study was the Sobol global sensitivity analysis (GSA) method, which provides quantitative results and incorporates the entire range of parameter values. With this method, all input parameters are varied simultaneously, accounting for non-linearities and capturing parameter interaction effects. In this study, total effect Sobol Sensitivity Indices have been computed, which are based on the ANOVA decomposition as extensively discussed in the literature [33–35].

The Sobol GSA was applied to the bottom-up model with a total of 46 input parameters to be examined. The GSA has been performed on the parameters as initially estimated, as opposed to values obtained from literature. This includes 30 enzyme concentrations, eight dissociation constants, seven turnover rates and one Hill coefficient. The enzyme concentration governing the outlet rate towards glycolysis has been excluded from the GSA as it accounts for between 95.8% and 97.6% of total glucose flux into the cell at any given time. Those values are in agreement with Chen *et al.*, who calculated the fraction of glucose, going towards the nucleotide sugar metabolic network to be 3.52% for CHO cells [27] based on previous experimental work by Goudar *et al.* [36]. This parameter is known to be important and was expected to dwarf others, as was confirmed by a preliminary GSA study. The remaining 46 parameters created a very large parameter space and thus, a very large sample set was required in order to map the model behavior accurately resulting in computationally very expensive calculations. In order to reduce calculation times, a metamodel was created using SobolHDMR v2.0, a general purpose metamodeling software. The program is based on a number of Random Sampling-High dimensional model representation (RS-HDMR) methods. The original RS-HDMR method is presented by Li *et al.* [37,38] and Li and Rabitz [39] while the improved techniques are described in [40,41]. All of the implemented techniques use Quasi-Monte Carlo sampling based on Sobol sequences. Supplied with “black-box” data of input parameter values with a sample set of 131,072 (2^17^) and corresponding model outputs a metamodel was created upon which the GSA was performed thus, greatly reducing computational cost.

The GSA was performed for all seven nucleotide sugars of interest at time points 20, 40, 60, 80 and 120 h corresponding to early-exponential, mid-exponential, late-exponential, stationary and decline growth phases, respectively. Indices greater than 0.036, *i.e.*, the lowest experimental error in NSD concentration measurements, have been deemed significant.

## 3. Model Performance and Discussion

**Figure 4.**
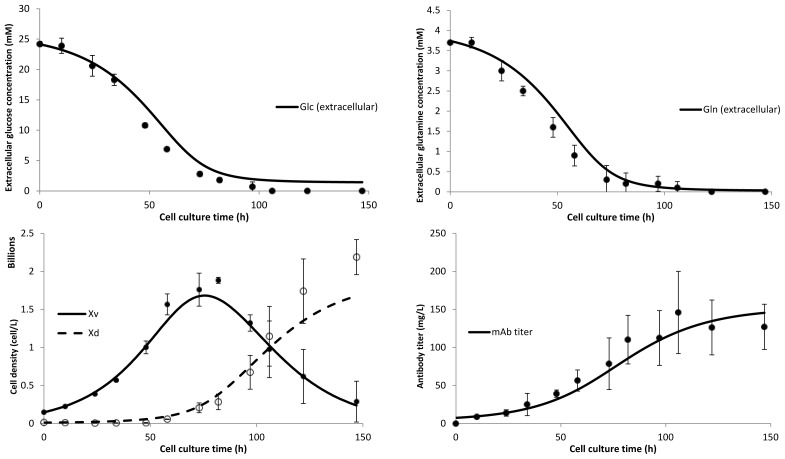
Experimental data compared with generated fits from the dynamic Monod growth model. Fits for extracellular glucose concentration (**top left**), extracellular glutamine concentration (**top right**), viable and dead cell density (**bottom left**) and antibody titer (**bottom right**).

The dynamic Monod kinetics growth model was able to describe the consumption of glucose and glutamine as well as cell growth and cell death (Figure 4). Interestingly, the model equation describing mAb production was a function of cell density only, but was still able to describe mAb titer well, indicating that mAb productivity was independent of the specific cell growth rate.

**Figure 5.**
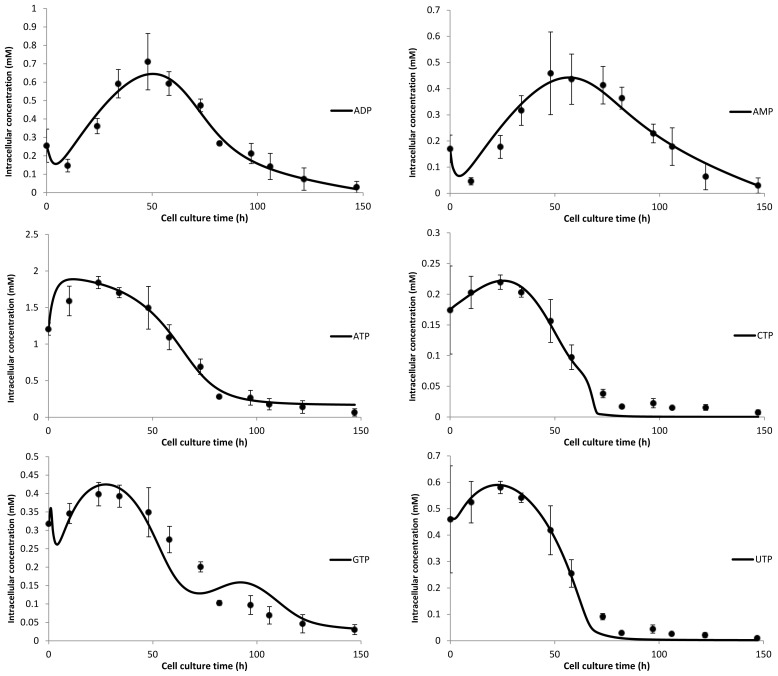
Experimental data compared with generated fits from the nucleotide model. Fits for intracellular ADP concentration (**top left**), intracellular AMP concentration (**top right**), intracellular ATP concentration (**center left**), intracellular CTP concentration (**center right**), intracellular GTP concentration (**bottom left**) and intracellular UTP concentration (**bottom right**).

Figure 5 depicts the fits of the simple nucleotide model as compared with the experimentally determined intracellular nucleoside concentrations. The model based on the reduced network was able to produce satisfactory fits. Some oscillatory behavior can be observed, most notably with the GTP model output. This effect is most likely due to the assumption that the flux of nucleotides out of the system is a function of growth only. However, to improve upon this fit would require an extensive analysis of the intracellular fluxes of the nucleotides involved and thus, decoupling the outlet flux from the cell growth term.

**Figure 6.**
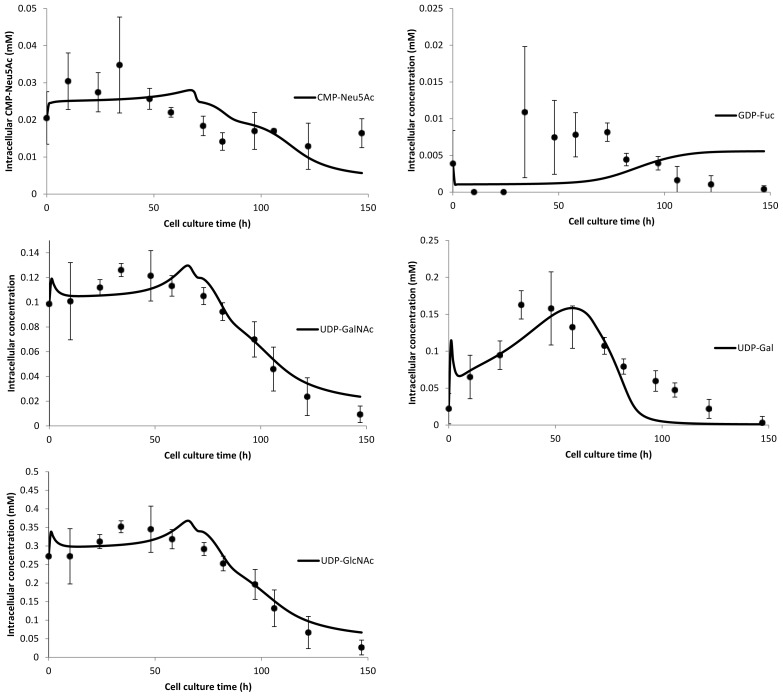
Experimental data compared with generated fits from the mechanistic bottom-up approach nucleotide sugar synthesis model. Fits for intracellular CMP-Neu5AC concentration (**top left**), intracellular GDP-Fuc concentration (**top right**), intracellular UDP-GalNAc concentration (**center left**), intracellular UDP-Gal concentration (**center right**) and intracellular UDP-GlcNAc concentration (**bottom left**).

The main focus of the presented work is the mechanistic bottom-up nucleotide sugar synthesis model, which was able to produce good fits for all species except for GDP-Fuc (Figure 6). The difficulties in estimating GDP-Fuc concentrations stem from its comparatively low intracellular concentration and, thus, the relatively large uncertainty in its measurements (including concentrations below the detection limit for some time points). Additionally, due to the lack of dynamic GDP-Man data, estimations of dissociation constants and enzyme concentrations of the reaction steps leading up to GDP-Fuc do not restrict the parameter space sufficiently to arrive at a satisfying result. Like the nucleotide model, the NSD outlet fluxes are a function of the cell growth rate. To improve on the fits, a detailed flux balance analysis will be required to constrain the *in vivo* metabolic network in a more physiologically accurate way and therefore depict its behavior more accurately *in silico*. The size of the metabolic network and the various intra-network interactions pose an additional challenge to the estimation of parameters as well as the design of experiments to improve parameter fit. Since many parameters were shown to have insignificant impact on model outputs, this presents scope for a formal model reduction to tackle the aforementioned difficulties. All estimated parameters are summarized in Appendix 3. While extensive quantitative proteomic [42,43] as well as transcriptomic [44] maps for mammalian species are beginning to appear, there is no data available for absolute enzyme concentrations within the NSD metabolic network. Additionally, the uncertainty in turnover rate data makes a comparison of estimated enzyme concentrations with literature data not feasible at this point.

**Figure 7.**
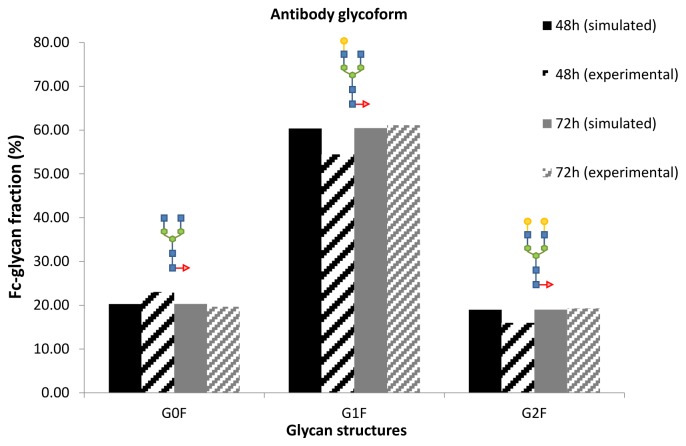
Simulated and experimentally determined distribution of the cumulative *N*-linked glycoform of the antibody Fc region for two time points during cell culture.

The output from the nucleotide sugar donor model, *i.e.*, the NSD concentrations, were fed into a model describing the cumulative *N*-linked glycosylation of the antibody Fc region as presented by del Val *et al.* [17] in order to estimate the resulting glycan distribution on the product. The results compare well with the measured data for product glycosylation, as shown in Figure 7. The glycan data for time points 48 and 72 h of the cell culture corresponding to exponential and stationary growth phases, respectively, are shown compared to the model simulation results. The glycan distribution is estimated by the model in sufficient accuracy. The small deviations observed suggest that the experimentally observed changes during this period may not be due to changes in nucleotide sugar concentration alone, but possibly also caused by variations in the levels of glycosylatransferases [28]. A further indication that the degree of galactosylation is not a strong function of UDP-Gal during the given time period is the very low *K*_M_ value of 0.0024 mM for the UDP-Gal Golgi transporter [45]. This is well below the measured and estimated UDP-Gal concentrations and indicates saturation of the transport protein until later stages of the cell culture.

## 4. Materials and Methods

### 4.1. Cell Culture, Metabolite Monitoring and Antibody Quantification

The mouse hybridoma HFN7.1 cell line (ATCC CRL-1606™, Teddington, UK) producing an IgG1 antibody against fibronectin from human plasma was used in this study. The cells were maintained in suspension cultures in 4.5 g/L glucose DMEM (Gibco, Paisley, UK) supplemented with 10% fetal bovine serum (Sigma-Aldrich, Gillingham, UK) at 37 °C and 5% CO_2_ on an orbital shaking platform rotating at 125 rpm. Subculture was performed every three days and new cultures were seeded at a density of 2 × 10^5^ cells/mL. Experiments were performed in 2 L vented Erlenmeyer flasks with a working volume of 400 mL. Cell concentration was determined using a Neubauer ruling haemocytometer and viability was estimated by the trypan blue dye exclusion method using light microscopy. Extracellular glucose, glutamine and lactate concentrations were measured using the BioProfile 400 (Nova Biomedical, Runcorn, UK). The antibody concentration in the supernatant was determined using an indirect sandwich enzyme-linked immunosorbent assay (ELISA) as described by Kontoravdi *et al.* [32].

### 4.2. Intracellular Nucleotide and Nucleotide Sugar Extraction

Cell culture samples were centrifuged at 800 rpm and the resulting cell pellet was resuspended in 200 μL of 0.5 M perchloric acid solution (Sigma-Aldrich, Gillingham, UK). The solution was incubated on ice for 10 min before centrifugation (4 °C, 10,000× *g*, 5 min). The supernatant was incubated with 40 μL of potassium hydroxide (Sigma-Aldrich, Gillingham, UK) on ice for a further 10 min, after which it was again centrifuged (4 °C, 10,000× *g*, 5 min). The final supernatant sample was filtered through a 0.22 μm syringe filter and briefly stored at 5 °C prior to analysis. Quenching with 0.9% *w*/*v* sodium chloride prior to extraction, as described by Dietmair *et al.* [46] has been investigated previously and shown not to affect NSD recovery, thus, quenching was deemed to be unnecessary [47].

### 4.3. Characterization of Intracellular Nucleotides and Nucleotide Sugars

An optimized high-performance anion-exchange chromatography (HPAEC) method as described in [47] was used for nucleotide sugar and nucleotide quantification. HPAEC elutions were performed with gradients of 1.5 M sodium acetate solution (Sigma-Aldrich, Gillingham, UK) in 3 mM potassium hydroxide (Sigma-Aldrich, Gillingham, UK) with a maximum ion concentration of 1 M sodium acetate using a CarboPac PA1 column (Dionex, Bannockburn, IL, USA). The applied method was able to resolve and detect six nucleotide sugar standards namely cytidine monophosphate *N*-acetylneuraminic acid (CMP-Neu5Ac), guanosine diphosphate fucose (GDP-Fuc), guanosine diphosphate mannose (GDP-Man), uridine diphosphate *N*-acetylgalactosamine (UDP-GalNAc), uridine diphosphate glucose (UDP-Glc), uridine diphosphate *N*-acetylglucosamine (UDP-GlcNAc) as well as adenine diphosphate (ADP), adenine monophosphate (AMP), adenine triphosphate (ATP), cytidine triphosphate (CTP), guanosine triphosphate (GTP) and uridine triphosphate (UTP) within a 20 min run.

### 4.4. Glycan Purification and Analysis

Antibody samples were purified using the Proteus Protein G antibody purification kit (Pro-Chem, Littleton, MA, USA) according to the manufacturer’s instructions. The purified antibody samples were lyophilized in a ModulyoD freeze dryer (Thermo Fisher Scientific, Waltham, MA, USA) prior to reduction and carboxymethylation, as described previously [48,49]. Briefly, reduction and carboxymethylation consisted of incubating the sample at 37 °C for 1 h in 1 mL of 0.6 M Tris-HCl buffer (Pierce, Rockford, IL, USA), pH 8.5, supplemented with 2 mg/mL dithioreitol (Sigma Aldrich, Gillingham, UK). Then 100 μL of 12 mg/mL iodoacetic acid (Sigma Aldrich, Gillingham, UK) in 0.6 M Tris-HCl buffer, pH 8.5 was added and incubating in the absence of light for 2 h at 37 °C. The product was dialysed against 4.5 L ammonium bicarbonate buffer, pH 7.7 for 24 h with regular buffer changes. The reduced and carboxymethylated samples were lyophilized once more, subjected to trypsin (TPCK treated bovine pancreas trypsin, EC 3.4.21.4) digestion and purified using reverse phase Sep-Pak C18 cartridges (Waters, Elstree, UK) as described previously [48,49]. Peptide-bound *N*-glycans were released by incubating 200 μL of the purified tryptic digest with 2.5 units of recombinant Peptide-*N*-glycosidase F (PNGase F, EC 3.5.1.52) from *E.coli* (Roche Applied Science, Burgess Hill, UK) for 24 h at 37 °C. The samples were lyophilized and dissolved in 200 μL of 5% (*v*/*v*) acetic acid (Romil, Cambridge, UK) and purified using the 1-propanol/5% acetic acid system on Sep-Pak C18 cartridges as described in [49]. The resulting 5% (*v*/*v*) acetic acid solutions were pooled and lyophilized in preparation for *N*-glycan NaOH permethylation [48,49]. The latter procedure was performed as described in [48,49]. The resulting permethylated samples were lyophilized and subsequently purified using the methanol/acetonitrile C18 Sep-Pak system [47]. The acetonitrile fractions were collected and lyophilized prior to dissolution in 10 μL methanol (Romil, Cambridge, UK). 2 μL of this methanol solution were mixed in a 1:1 ratio with 20 mg/mL 2,5-dihydrobenzoic acid (Fluka, Gillingham, UK) dissolved in 70% (*v*/*v*) aqueous methanol and loaded onto a 100-well MALDI metal plate [49]. The MALDI plate was placed under vacuum for ~20 min, and when completely dry, was loaded into the mass spectrometer for analysis.

MALDI-MS was performed on a Voyager-DE STR MALDI workstation (Perspective Biosystems, Paisley, UK) equipped with delayed extraction technology. The machine was set in positive reflectron mode and data was acquired using the Voyager 5 Instrument Control Software. Data was processed using Data Explorer 4.9 software (Applied Biosystems, Darmstadt, Germany), where baseline correction and noise filtering (correction factor of 0.7) was performed. The mass spectra were then transferred to the GlycoWorkBench software [50,51] for peak assignment and relative quantification of the observed glycans.

## 5. Conclusions

The holistic modelling framework presented is a first step towards quantifying the impact of extracellular nutrient concentrations on the glycoform of biotherapeutics through capturing changes in the nucleotide and nucleotide sugar metabolism. A closed-loop *in silico* platform could ultimately be used for the optimisation of glycosylation in biotherapeutics. More work is required to assess how the availability of nucleotides and the addition of another, or even a combination of, different carbon sources will impact the metabolism of nucleotide sugars. However, in order to capture the *in vivo* behavior more accurately *in silico*, a flux balance approach is required to constrain model inlets and outlets more accurately, particularly with respect to the role of amino acids in the *de novo* synthesis of nucleotide sugars and, more importantly, nucleotides. By extending the dynamic growth model to capture changes in extracellular amino acid concentrations and using those changes as dynamic inputs for a flux balance model, the dynamic nature and predictive capability of the present modelling framework can be significantly enhanced. Furthermore, extending the modelling platform to other mammalian cell lines, most notably Chinese hamster ovary cells (CHO), and products could produce a more versatile and industrially relevant modelling platform for *in silico* optimisation of the glycosylation process.

## Figures and Tables

**Table 1.** Frequency of sugar species occurrence in glycans of mature human B-cells.

Glycan type	GlcNAc	GalNAc	Man	Gal	Neu5Ac	Fuc
*N*-linked glycan	2.896	0	5.813	0.759	0.516	0.332
*O*-linked glycan	0.156	1	0	1.156	1.543	0
Glycan average (*N*_NSD,glyc_)	1.579	0.481	3.018	0.950	1.010	0.173

**Figure A1.**
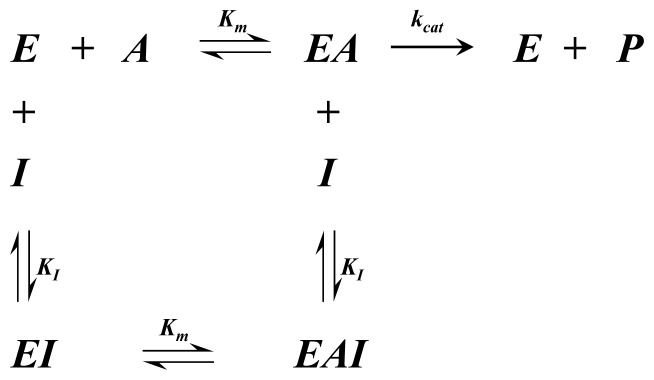
Single substrate Michaelis-Menten kinetics and competitive inhibition of species A through I.

**Figure A2.**
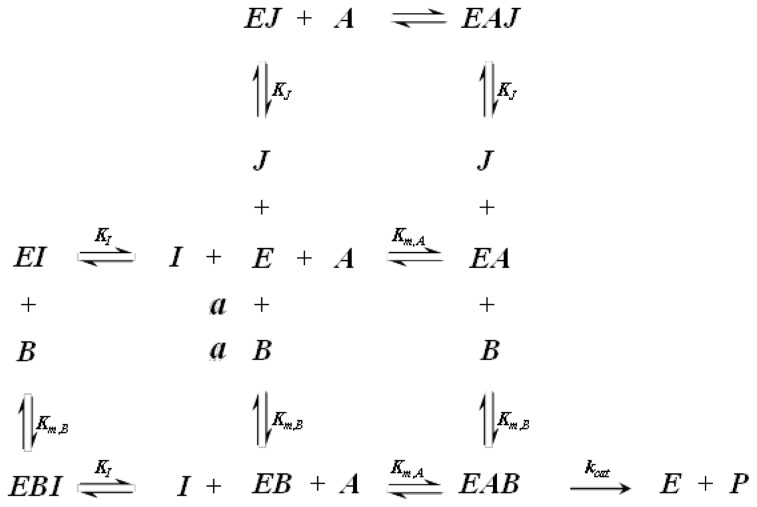
Random order bi-bi kinetics and competitive inhibition of species A through I and species B competing with J.

**Figure A3.**
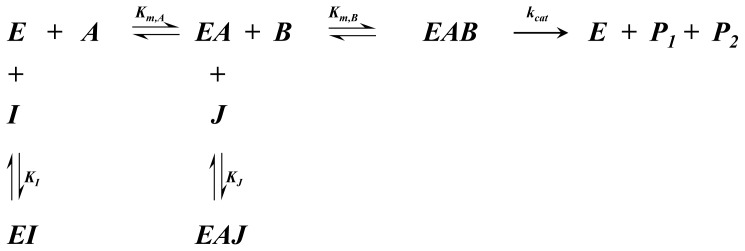
Ordered bi-bi kinetics and competitive inhibition of species A through I and species B competing with J.

**Figure A4.**
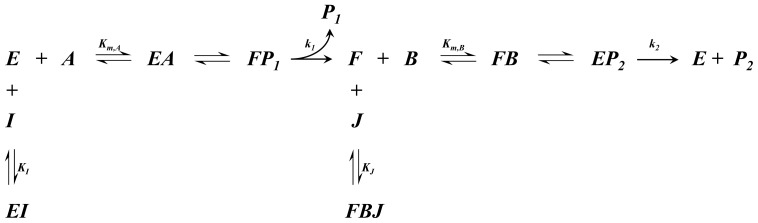
Ping-pong bi-bi kinetics and competitive inhibition of species A through I and species B competing with J.

**Figure A5.**
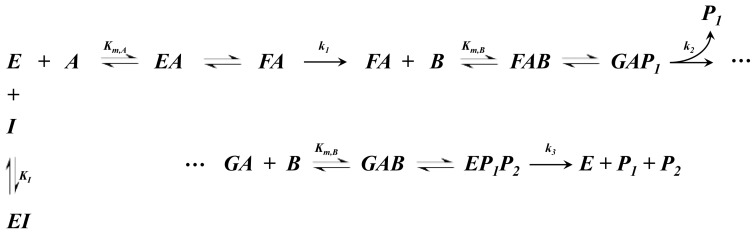
Ping-pong ter-ter kinetics and competitive inhibition of species A through I.

**Figure A6.**
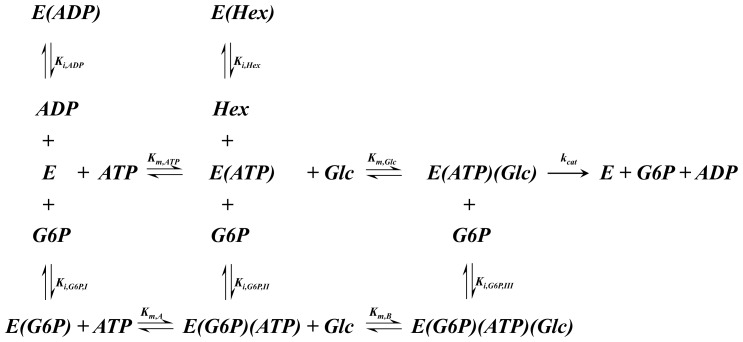
Hexokinase reaction scheme including competitive inhibition of species ATP through ADP, Glucose competing with other hexose species and un-competitive inhibition through Glucose-6P.

## Appendix 1—Enzyme Mechanisms

### A1.1. Single Substrate Michaelis-Menten Rate Equation

Based on the listed assumptions the following reaction scheme for single substrate Michaelis-Menten kinetics was set up:

Based on this reaction scheme and the previously described assumptions, the following rate of reaction equations were derived including inhibitory enzyme kinetics for competitive and non-competitive inhibition:

**Table:**

Equation (1) Rate of reaction for Single substrate Michaelis-Menten kinetics	$v=\frac{d[P]}{dt}=\frac{k_{cat}[E_{0}][A]}{K_{m}+[A]}$
Equation (2) Rate of reaction for Single substrate Michaelis-Menten kinetics including competitive inhibition of species A	$v=\frac{d[P]}{dt}=\frac{k_{cat}[E_{0}][A]}{K_{m}\left(1+\sum_{i=1}^{NI}\frac{\left[I_{i}\right]}{K_{I,i}}\right)+[A]}$
Equation (3) Rate of reaction for Single substrate Michaelis-Menten kinetics including non-competitive inhibition	$v=\frac{d[P]}{dt}=\frac{k_{cat}[E_{0}][A]}{(K_{m}+[A])\;\left(1+\frac{\left[I\right]}{K_{I}}\right)},$

### A1.2. Random Order Bi-Bi Kinetics

Based on the previous assumptions the following reaction scheme for random order bi-bi kinetics was set up:

Based on this reaction scheme and the previously described assumptions, the following rate of reaction equations were derived including inhibitory enzyme kinetics for competitive and non-competitive inhibition:

**Table:**

Equation (4) Rate of reaction for random order bi-bi kinetics	$v=\frac{d[P]}{dt}=\frac{k_{cat}[E_{0}][A][B]}{K_{m,A}K_{m,B}+K_{m,A}[B]+K_{m,B}[A]+[A][B]}$
Equation (5) Rate of reaction for random order bi-bi kinetics including competitive inhibition of species A and B	
$v=\frac{d[P]}{dt}=\frac{k_{cat}[E_{0}][A][B]}{K_{m,A}K_{m,B}\left(1+\sum_{i=1}^{NI}\frac{\left[I_{i}\right]}{K_{I,i}}+\sum_{j=1}^{NJ}\frac{\left[J_{j}\right]}{K_{J,j}}\right)+K_{m,A}[B]\;\left(1+\sum_{i=1}^{NI}\frac{\left[I_{i}\right]}{K_{I,i}}\right)+K_{m,B}[A]\;\left(1+\sum_{j=1}^{NJ}\frac{\left[J_{j}\right]}{K_{J,j}}\right)+[A][B]}$	
Equation (6) Rate of reaction for random order bi-bi kinetics including non-competitive inhibition	
$v=\frac{d[P]}{dt}=\frac{k_{cat}[E_{0}][A][B]}{(K_{m,A}K_{m,B}+K_{m,A}[B]+K_{m,B}[A]+[A][B])\;\left(1+\frac{\left[I\right]}{K_{I}}\right)},$	

### A1.3. Ordered Bi-Bi Kinetics

Based on the previous assumptions the following reaction scheme for ordered bi-bi kinetics was set up:

Based on this reaction scheme and the previously described assumptions, the following rate of reaction equations were derived including inhibitory enzyme kinetics for competitive and non-competitive inhibition:

**Table:**

Equation (7) Rate of reaction for ordered bi-bi kinetics	$v=\frac{d[P]}{dt}=\frac{k_{cat}[E_{0}][A][B]}{K_{m,A}K_{m,B}+K_{m,B}[A]+[A][B]}$
Equation (8) Rate of reaction for ordered bi-bi kinetics including competitive inhibition of species A and B	$v=\frac{d[P]}{dt}=\frac{k_{cat}[E_{0}][A][B]}{K_{m,A}K_{m,B}\left(1+\sum_{i=1}^{NI}\frac{\left[I_{i}\right]}{K_{I,i}}\right)+K_{m,B}[A]\;\left(1+\sum_{j=1}^{NJ}\frac{\left[J_{j}\right]}{K_{J,j}}\right)+[A][B]}$
Equation (9) Rate of reaction for ordered bi-bi kinetics including non-competitive inhibition	$v=\frac{d[P]}{dt}=\frac{k_{cat}[E_{0}][A][B]}{(K_{m,A}K_{m,B}+K_{m,B}[A]+[A][B])\;\left(1+\frac{\left[I\right]}{K_{I}}\right)},$

### A1.4. Ping-Pong Bi-Bi Kinetics

Based on the previous assumptions the following reaction scheme for ping-pong bi-bi kinetics was set up:

Based on this reaction scheme and the previously described assumptions, the following rate of reaction equations were derived including inhibitory enzyme kinetics for competitive and non-competitive inhibition:

**Table:**

Equation (10) Rate of reaction for ping-pong bi-bi kinetics	$v=\frac{d[P]}{dt}=\frac{k_{cat}[E_{0}][A][B]}{K_{m,A}[B]+K_{m,B}[A]+[A][B]}$
Equation (11) Rate of reaction for ping-pong bi-bi kinetics including competitive inhibition of species A and B	$v=\frac{d[P]}{dt}=\frac{k_{cat}[E_{0}][A][B]}{K_{m,A}[B]\;\left(1+\sum_{i=1}^{NI}\frac{\left[I_{i}\right]}{K_{I,i}}\right)+K_{m,B}[A]\;\left(1+\sum_{j=1}^{NJ}\frac{\left[J_{j}\right]}{K_{J,j}}\right)+[A][B]}$
Equation (12) Rate of reaction for ping-pong bi-bi kinetics including non-competitive inhibition	$v=\frac{d[P]}{dt}=\frac{k_{cat}[E_{0}][A][B]}{(K_{m,A}[B]+K_{m,B}[A]+[A][B])\;\left(1+\frac{\left[I\right]}{K_{I}}\right)},$

### A1.5. Ping-Pong Ter-Ter Kinetics

Based on the previous assumptions the following reaction scheme for ping-pong ter-ter kinetics was set up:

Based on this reaction scheme and the previously described assumptions, the following rate of reaction equations were derived including inhibitory enzyme kinetics for competitive and non-competitive inhibition:

**Table:**

Equation (13) Rate of reaction for ping-pong ter-ter kinetics including competitive inhibition of species A
$v=\frac{d[P]}{dt}=\frac{k_{cat}[E_{0}][A]{\left[B\right]}^{2}}{2K_{m,A}K_{m,B}[B]+2K_{m,B}[A][B]+K_{m,A}{\left[B\right]}^{2}\;\left(1+\frac{\left[I\right]}{K_{I}}\right)+{\left(K_{m,B}\right)}^{2}[A]+[A]{\left[B\right]}^{2}}$

### A1.5. Hexokinase Rate of Reaction Expression

The rate of reaction for Hexokinase cannot be described by any of the previously described rate expressions. This is due to its competitive inhibition of ADP with ATP, various Hexose sugar substrates competing (namely glucose, glucosamine, fructose and mannose) and un-competitive inhibition by glucose-6-phosphate. Based on previous assumptions a reaction scheme for the catalysis of glucose to glucose-6-phosphate can be represented as depicted in Figure 16. Based on this reaction scheme and the previously described assumptions, the following rate of reaction equations were derived:

**Table:**

Equation (14) Rate of reaction for hexokinase based on the above reaction scheme
$v=\frac{d[P]}{dt}=\frac{k_{cat}[E_{0}][ATP][Glc]}{K_{m,ATP}K_{m,Glc}\left(1+\frac{\left[ADP\right]}{K_{i,ADP}}+\frac{\left[G6P\right]}{K_{i,G6P,I}}\right)+[ATP]K_{m,Glc}\left(1+\frac{\left[G6P\right]}{K_{i,G6P,I}}+\frac{\left[G6P\right]}{K_{i,G6P,II}}+\Sigma \frac{\left[{Hex}_{i}\right]}{K_{i,Hex}}\right)+[ATP][Glc]\;\left(1+\frac{\left[G6P\right]}{K_{i,G6P,I}}+\frac{\left[G6P\right]}{K_{i,G6P,II}}+\frac{\left[G6P\right]}{K_{i,G6P,III}}\right)}$

### A1.6. Glycolysis

The rate of reaction has been described by Chen *et al.* and is given by Equation (16), where both the dissociation constant for Fructose-6-phosphate and the Hill coefficient, are a function of fructose-2,6-bisphosphate [27].

Equation (15) describing the rate of Fructose-6-P removal to glycolysis

$$v=\frac{d[P]}{dt}=\frac{k_{cat}[E_{0}]}{\left[1+{\left(\frac{K_{m,F6P}}{\left[F6P\right]}\right)}^{n}\right]\;\left(1+\frac{K_{m,ATP}}{\left[ATP\right]}\right)}$$

### A1.7. Hill Coefficients

The Hill equation is used to yield thermodynamic information about homotropic reactions and is a measure of cooperativity of the binding reaction. A value of 1 for *n* indicates non-cooperative behaviour and thus simple Michaelis-Menten kinetics, while values of less than 1 indicate negatively cooperative behaviour and values greater than 1 indicate positive cooperativity. Hill coefficients were obtained from literature for a number of reactions within the NSD metabolic network and are indicated in Table S1. Fractional occupation (θ) of an enzyme species for a single substrate reaction is described by Equation (15), where S denotes substrate concentration.

Equation (16) Hill equation describing the fractional occupation of an enzyme by its substrate

$$\theta =\frac{{\left[S\right]}^{n}}{K_{m}+{\left[S\right]}^{n}}$$

## Appendix 2—Relative Abundances of Activated Human B-Cell Glycans

The two tables shown below summarise the relative abundance of all *N*-linked and *O*-linked glycan species for activated human B-cells as determined from the relative MS intensities. The relative abundances were used to find an average *N*-linked and *O*-linked glycan composition and thus, an overall average glycan composition as described in part 3.3.

The compositions of *O*-linked and *N*-linked glycan structures of activated human B-cells are derived from putative glycan structures which are based on MALDI-TOF-MS molecular ion compositions, MALDI-TOF/TOF-MS fragmentation of molecular ions, GC-MS linkage analysis of partially methylated alditol acetates and the biosynthetic knowledge [49].

**Table A1.** Summary of the relative abundances of N-linked glycan structures used to obtain an average glycan structure.

Glycan structure	Sugar frequency per glycan (mol/mol)					

	Species abundance (%)	GlcNAc	Man	Gal	Fuc	CMP-Neu5Ac
	7.37	2	5			
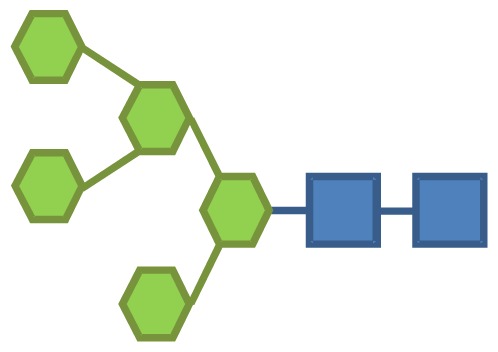
	10.86	2	6			
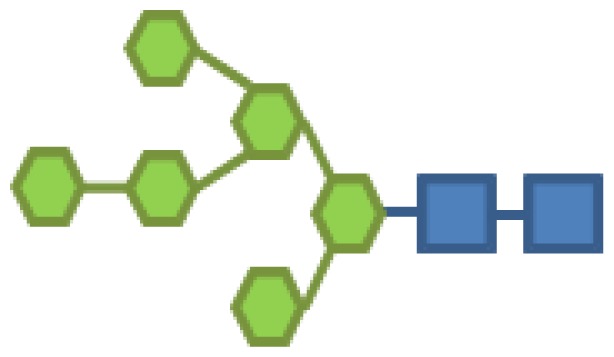
	1.75	4	3		1	
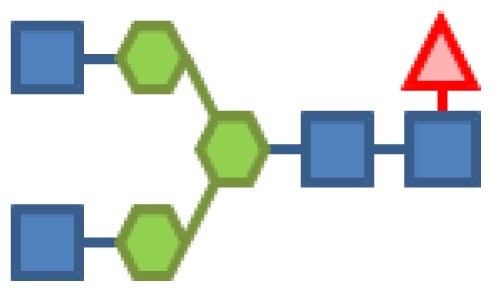
	13.40	2	7			
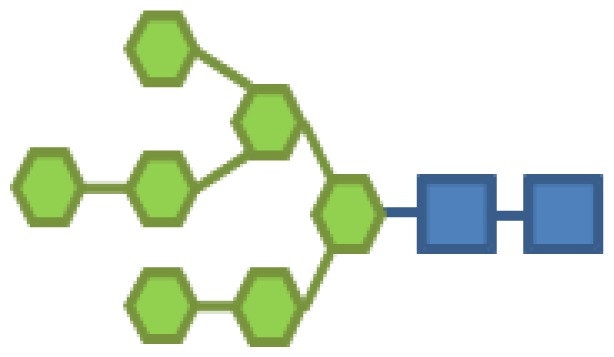
	1.75	4	3	1	1	
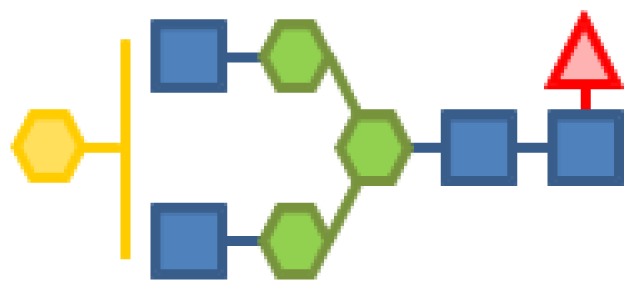
	14.85	2	8			
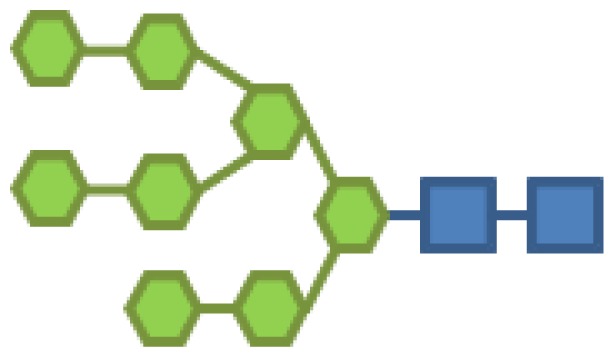
	0.69	4	3	2	1	
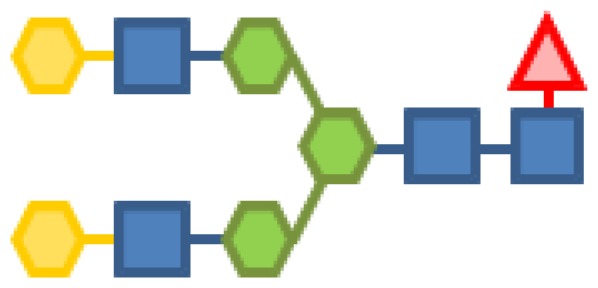
	0.47	5	3	1	1	
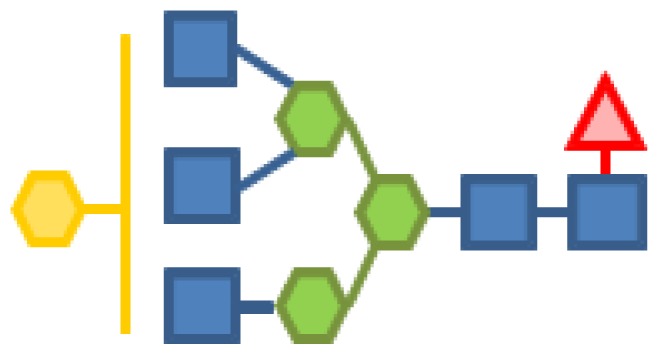
	17.69	2	9			
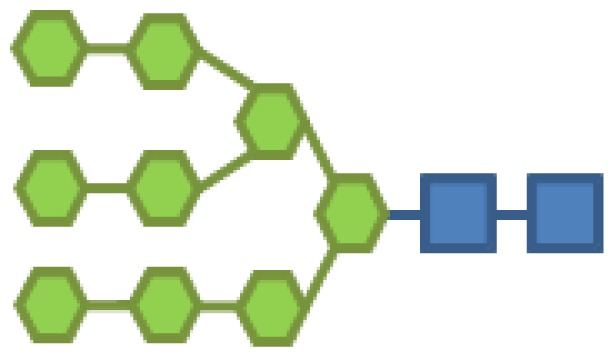
	1.87	4	3	2		1
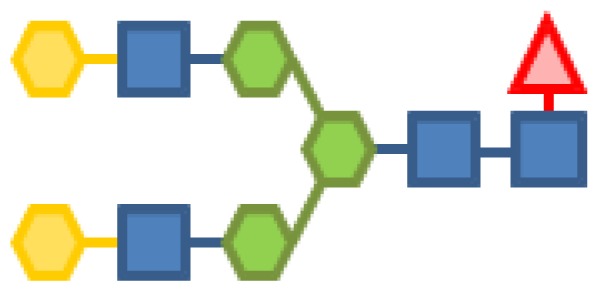
	0.44	5	3	2	1	
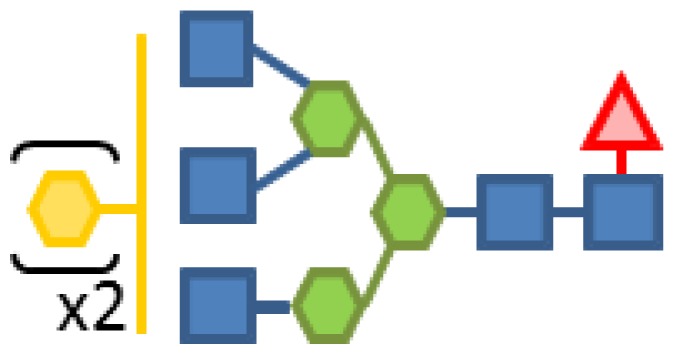
	2.97	4	3	2	1	1
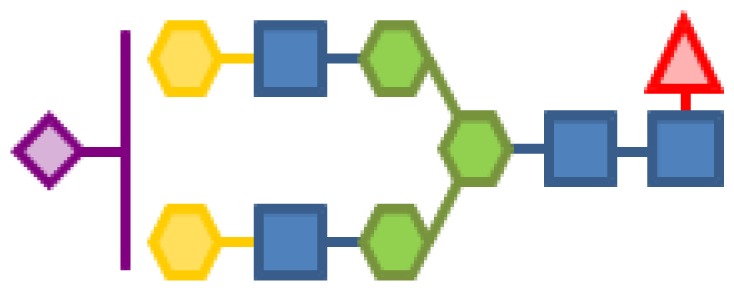
	0.49	4	3	2	2	1
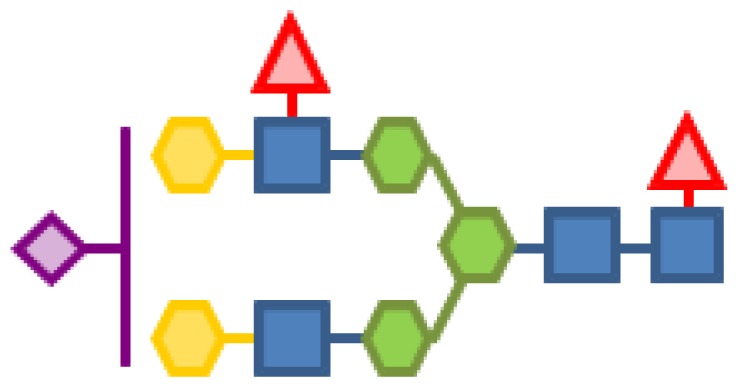
	1.23	4	3	2		2
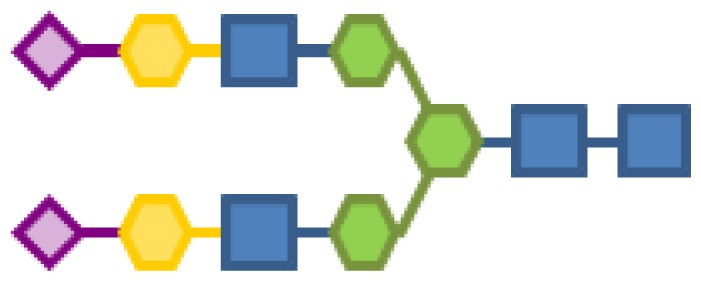
	10.78	4	3	2	1	2
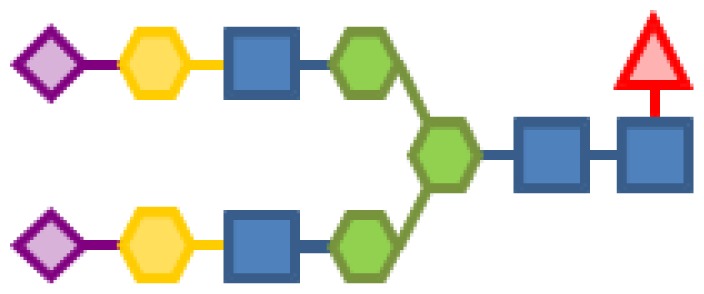
	2.59	5	3	3	1	1
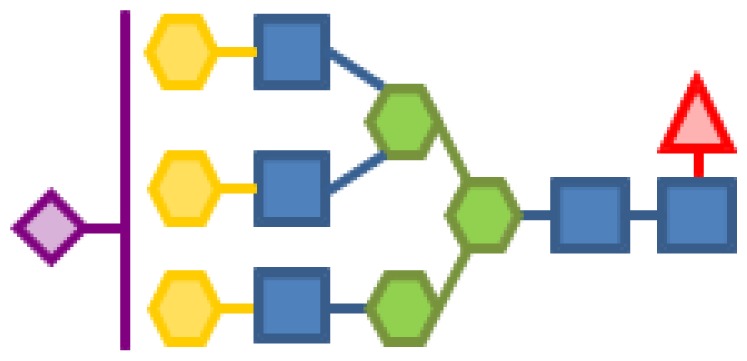
	5.03	5	3	2	1	2
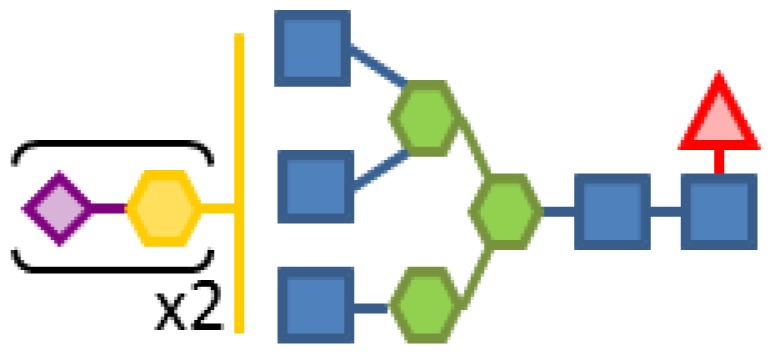
	0.34	6	3	3	1	1
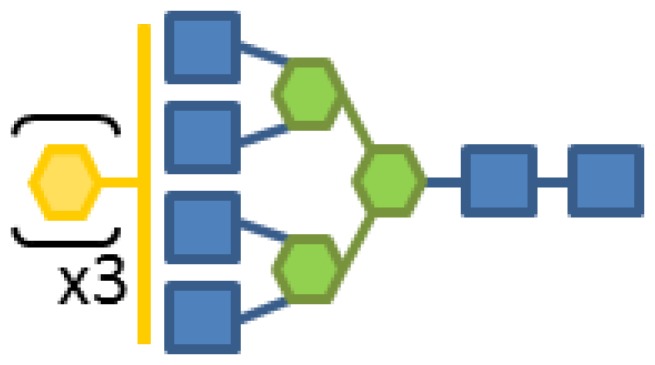
	1.35	5	3	3	1	2
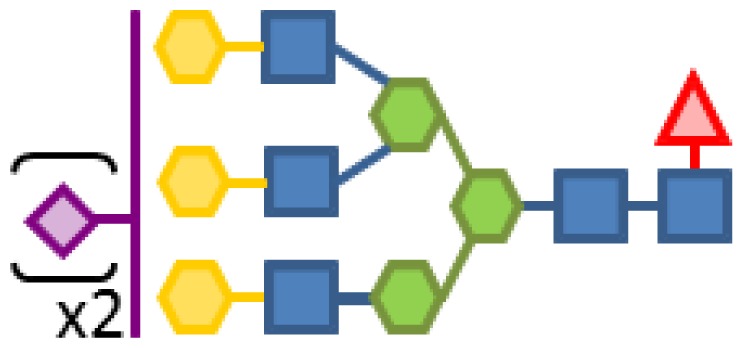
	0.59	6	3	4	1	1
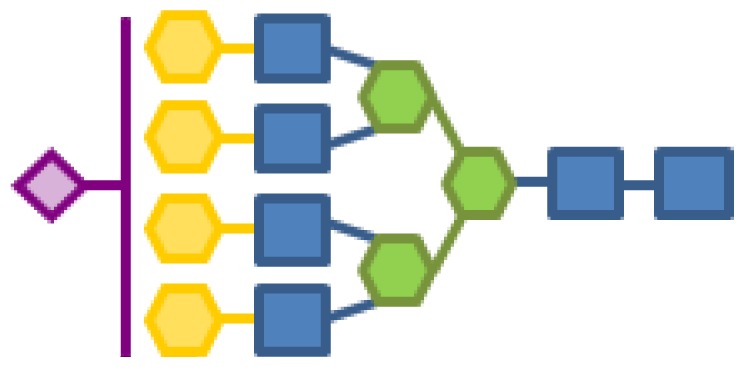
	0.12	6	3	3	1	2
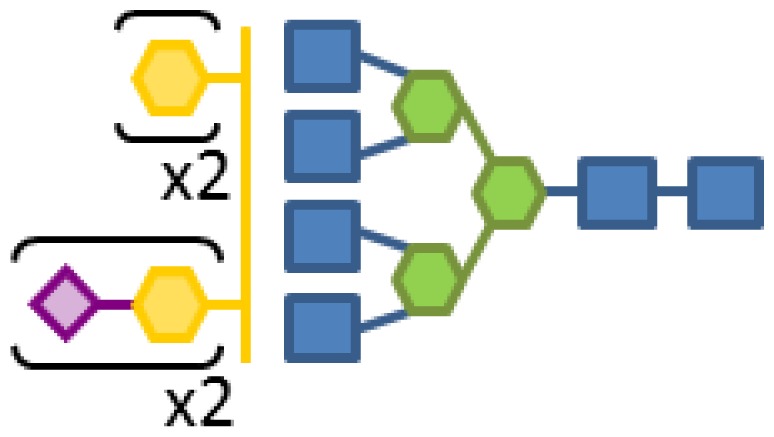
	0.10	7	3	4	1	1
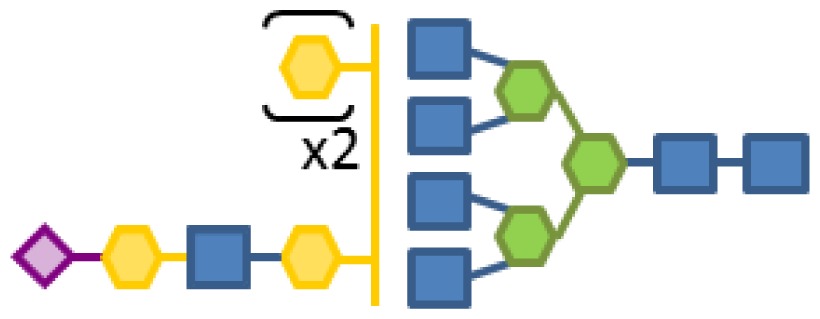
	0.31	5	3	3	1	3
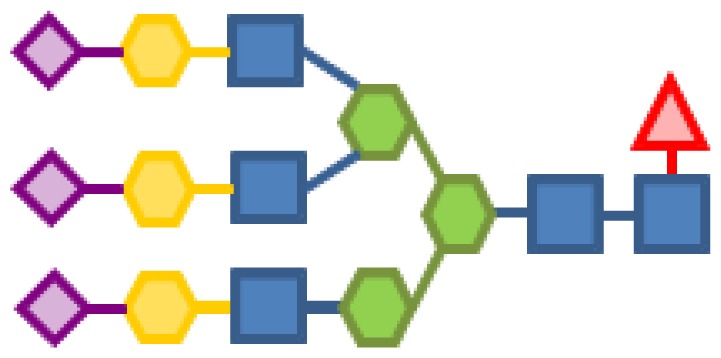
	0.68	6	3	4	1	2
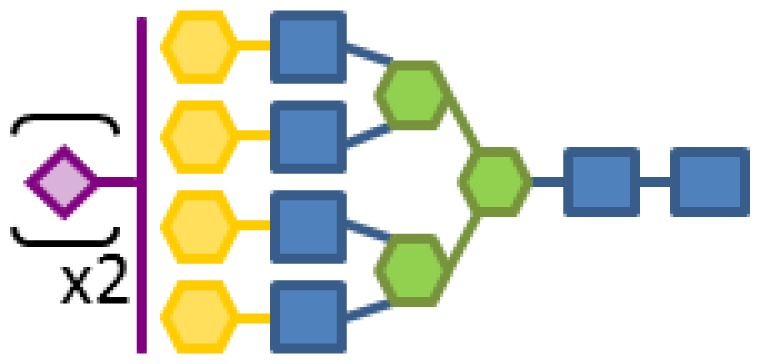
	0.10	7	3	5	1	1
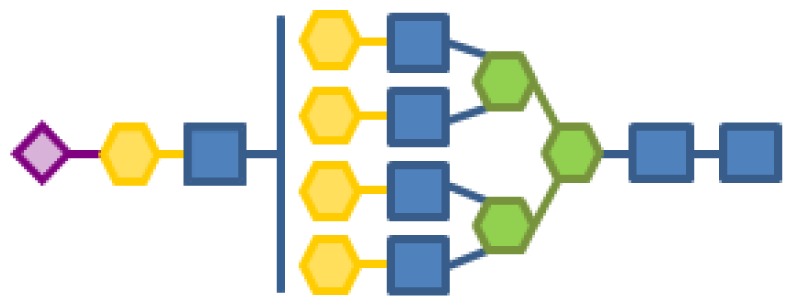
	0.09	7	3	4	1	2
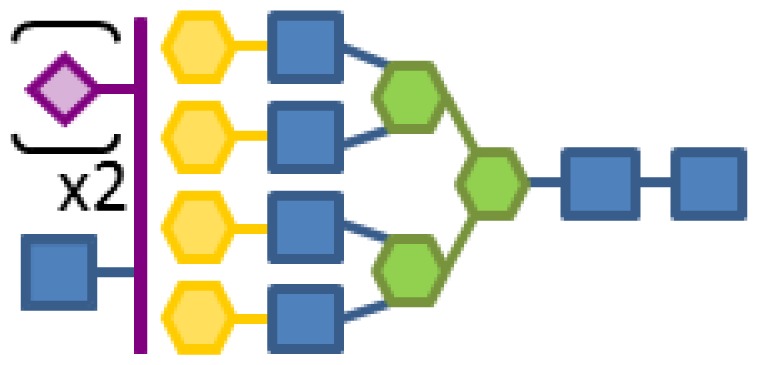
	0.18	6	3	4	1	3
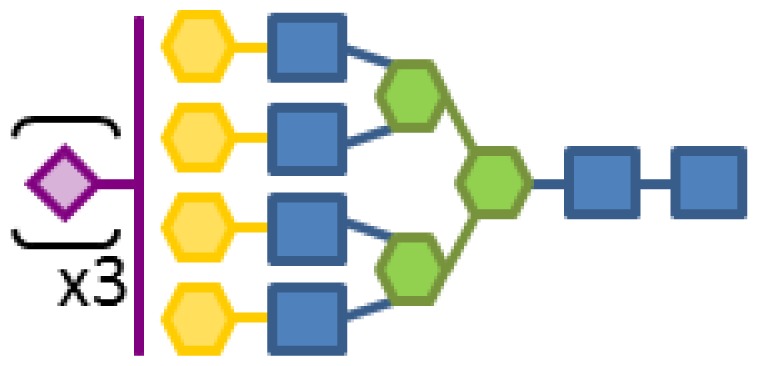
	0.36	7	3	5	1	2
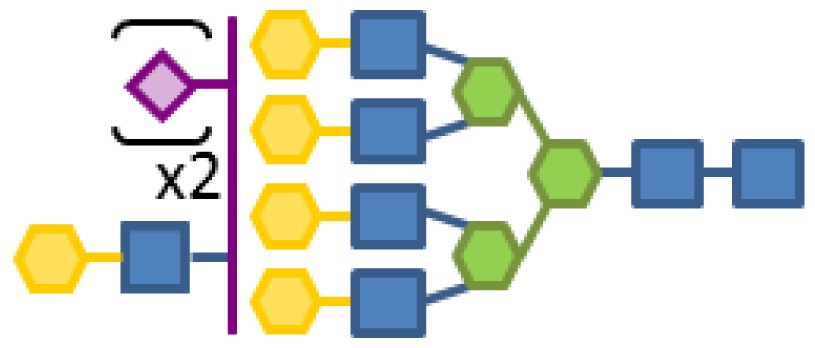
	0.09	7	3	5	1	3
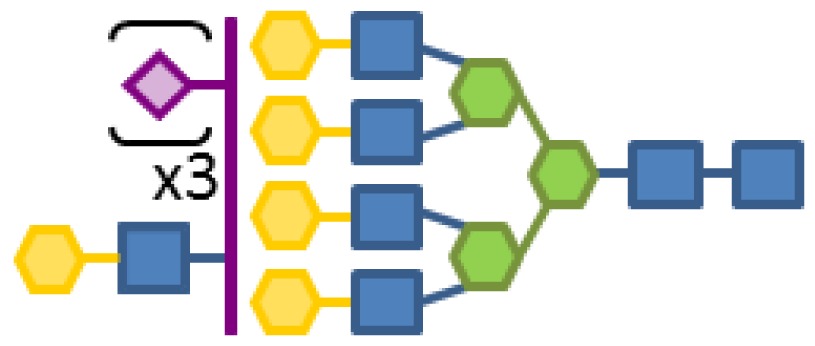
	0.09	8	3	6	1	2
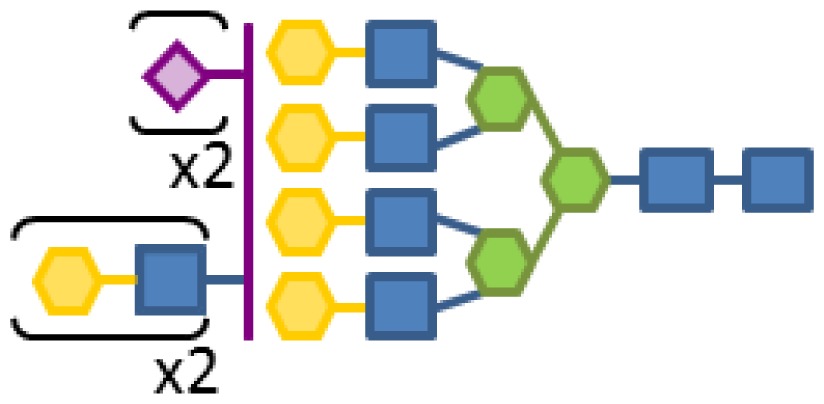

**Table A2.** Summary of the relative abundances of *O*-linked glycan structures used to obtain an average glycan structure.

Glycan structure	Sugar frequency per glycan (mol/mol)				

	Species abundance (%)	GlcNAc	GalNAc	Gal	CMP-Neu5Ac
	30.34		1	1	1
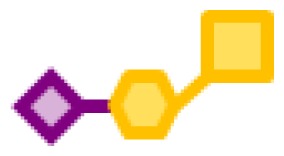
	2.18	1	1	2	
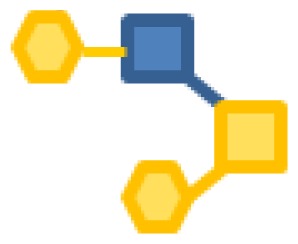
	53.64		1	1	2
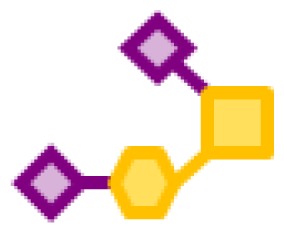
	11.42	1	1	2	1
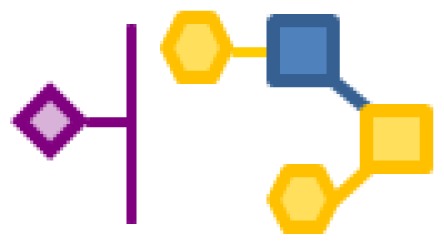
	0.77		1	1	3
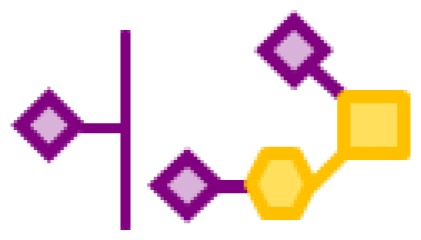
	1.27	1	1	2	2
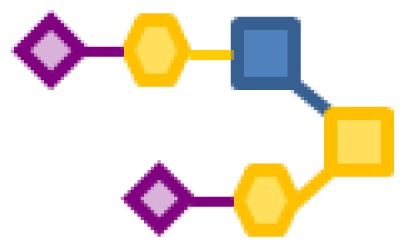
	0.38	2	1	3	1
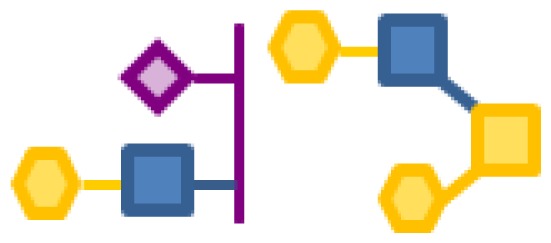

## Appendix 3—Estimated Parameter Values and Non-Nucleotide Species

### A3.1. NSD Metabolic Network Parameter Values

The first global sensitivity analysis results indicate that 22 of the 46 of the initially estimated parameters of the NSD metabolic network were significant. This allowed for a more targeted parameter estimation and the final estimated values are shown below.

**Table A3.** NSD metabolic netowrk parameter values.

Parameter name	Parameter value
E15a	2.5400E−05
E8a	0.0000E+00
E26b	0.0000E+00
E28a	0.0000E+00
E34a	0.0000E+00
E19a	6.4400E−06
E21a	6.8586E−06
Eglyc	7.0800E−05
Ki15a_GDPFuc	2.1200E−04
E29a	1.3313E−03
E1c	1.5096E−03
E1a	1.8477E−03
Km17a_Man6P	2.5300E−03
E23a	3.7138E−03
E12a	3.9000E−03
E32a	3.9000E−03
E14a	3.9000E−03
E16a	3.9000E−03
E20a	3.9000E−03
E2a	3.9000E−03
E37a	3.9000E−03
E3b	3.9000E−03
E40a	3.9000E−03
E4a	3.9000E−03
E4b	3.9000E−03
E17a	3.9532E−03
E22a	1.0000E−02
E3a	1.4167E−02
E38a	3.5128E−02
E13a	3.9000E−02
E31a	3.9000E−02
E33a	3.9000E−02
E5a	3.9000E−02
E6a	3.9000E−02
Km26a_Glc6P	9.3842E−02
Km22a_UDPGlc	2.0697E−01
Km7b_UDPGlcNAc	8.2129E−01
Gln_coef	1.0000E+00
n29a_CMPNeu5Ac	4.2000E+00
Ki_SA_Tra_UDPGlcNAc	6.8885E+00
Km19a_Fru6P	1.9700E+01
k5aB	6.0000E+02
Ki34a_CMPNeu5Gc	1.0000E+03
k26aF	2.0972E+03
k6aB	2.3900E+04
k17aF	7.0928E+04
k22aB	1.6151E+05
Ki29a_CMPNeu5Ac	5.2433E+05
k21aB	4.2900E+06
k21aF	6.1200E+06
k19aF	1.5900E+07
k27aF	1.1200E+08

**Table A4.** Cell dynamics model parameter values.

Parameter name	Parameter value
k_T_gln	3.3800E−06
Kd_Glc_ext	1.0051E−01
Kd_Gln_ext	1.1872E−02
Km_Glc_ext	2.6700E+00
Km_Gln_ext	1.2000E+00
mu_d_max_glc	3.9300E−01
mu_d_max_gln	6.2053E−02
mu_g_max	6.6745E−02
Y_ext_glc	9.1600E+07
Y_ext_gln	5.6400E+08
Y_mAb_mu	0.0000E+00
Y_mAb_Xv	1.1400E−09
Glc_in	0.0000E+00
Gln_in	0.0000E+00
F_in	0.0000E+00
F_out	0.0000E+00

**Table A5.** Nucleotide model parameter values.

Parameter name	Parameter value
Kdf_10_Gln	2.6356E+00
Kdf_11_Glc	2.1296E+00
Kdf_11_Gln	1.3425E+00
Kdf_12_ATP	1.1302E+01
Kdf_13_ADP	3.9892E−04
Kdf_13_Glc	4.7469E+00
Kdf_14_ADP	2.5000E+01
Kdf_15_AMP	1.1312E+01
Kdf_15_Glc	2.3025E+00
Kdf_8_Glc	1.2067E+00
Kdf_8_Gln	2.4832E+00
Kdf_9_Gln	2.1804E+00
Kdf_9_UTP	5.0374E−03
Kdout_ATP	1.0000E−03
Kdout_CTP	1.0000E−03
Kdout_GTP	1.0000E−03
Kdout_UTP	1.0000E−03
kf_10	7.6621E+00
kf_11	6.7500E+00
kf_12	3.2344E+00
kf_13	2.1454E−01
kf_14	1.1649E+02
kf_15	8.6900E+01
kf_8	7.9486E+00
kf_9	1.3393E+00

**Table A6.** *N*-linked antibody constant region glycan parameters.

Parameter name	Parameter value
KdiFucTA	0.0000E+00
KdiFucTB	0.0000E+00
KdiGalTa1A	1.0709E+02
KdiGalTa1B	7.2051E+00
KdiGalTa2A	3.4573E+01
KdiGntII	7.7067E+01

### A3.2. Non-NSD Species

In addition to the 34 sugars and nucleotide sugars there are 16 further intracellular species, which play a role in the catalysis of the NSD metabolic network as co-substrates, activators and inhibitors. Data was obtained from literature as reported by Williamson *et al.* and is listed in Table A5, where all concentrations are assumed to remain constant throughout the investigation [52].

**Table A7.** Non-nucleotide sugar species and their intracellular concentrations.

Intracellular species	Intracellular conc. (mM)	Source tissue
Acetyl Coenzyme A (**ACoA**)	0.029	Rat liver
Coenzyme A (**CoA**)	0.13	Rat liver
Glucose-1,6-biphosphate (**Glc16PP**)	0.014	Mouse liver
Nicotinamide adenine dinucleotide (**NAD**)	0.76	Rat liver
Nictotinamide adenine dinucleotide phosphate (**NADP**)	0.067	Rat liver
Nictotinamide adenine dinucleotide phosphate, reduced (**NADPH**)	0.30	Rat liver
Phosphoenolypyruvic acid (**PEP**)	0.11	Mouse liver
Inorganic phosphate (**PPi**)	3.37	Rat liver
Pyruvate (**Pyr**)	0.18	Mouse liver
